# Outcomes following intraoperative rupture of cerebral aneurysms during microsurgical clipping: a systematic review and meta-analysis

**DOI:** 10.1007/s10143-026-04221-0

**Published:** 2026-03-18

**Authors:** Brooklyn Brekke-Kumley, Kiana Yeganeh, Mackenzie Fox, Kristin Cler, Michael T. Lawton, Ali Tayebi Meybodi

**Affiliations:** 1 Rocky Vista University, Montana College of Osteopathic Medicine, Billings, MT USA; 2 Ponce Health Sciences University, School of Medicine, Ponce, Puerto Rico; 3 Department of Neurosurgery, Barrow Neurological Institute, Phoenix, AZ USA; 4 Department of Neurological Surgery, Rutgers New Jersey School of Medicine, Newark, NJ 08901 USA

**Keywords:** Intraoperative rupture, Intracranial aneurysm, Microsurgical clipping, Functional outcome, Mortality

## Abstract

Background: Intraoperative aneurysm rupture (IOR) during microsurgical clipping remains one of the most feared intraoperative complications, reported in approximately 7–35% of cases. IOR can precipitate abrupt hemodynamic instability, cerebral swelling, and potentially devastating neurological decline, mostly due to blind clipping of poorly seen perforators. This systematic review and meta-analysis aimed to quantify the association between IOR and poor neurological outcome and mortality, and to examine how these outcomes vary by aneurysm size and location. Methods: A comprehensive search of PubMed and Embase identified studies evaluating patients undergoing microsurgical clipping that reported IOR status and postoperative outcomes using the modified Rankin Scale (mRS) or Glasgow Outcome Scale (GOS). 19 studies met the inclusion criteria; thirteen provided comparative data for pooled analysis. Random-effects models were used to estimate pooled risk ratios (RR) for poor outcome (mRS 3–6 or GOS 1–3) and mortality. Pooled proportions of poor neurologic outcome among IOR cases were stratified by aneurysm size and location. Results: IOR was associated with a significantly higher risk of poor outcome (RR 1.67, 95% CI 1.34–2.09; I² = 59.6%; p < .001) and mortality (RR 2.11, 95% CI 1.37–3.25; I² = 42.8%; p < .001). The pooled poor-outcome rate among IOR cases was 36.6% (95% CI 29.0–44.9%). Poor-outcome proportions increased with aneurysm size: <7 mm, 15.2%; 7–12 mm, 29.3%; 13–24 mm, 62.5%; ≥25 mm, 81.8%. MCA and AComA aneurysms demonstrated similar rates (37.4% vs. 36.2%). Conclusions: IOR during microsurgical clipping confers a statistically and clinically significant increase in poor neurological outcomes and mortality. Continued refinement of intraoperative strategies and preventive techniques remains essential to mitigate IOR risk and improve patient prognosis.

**Supplementary Information:**

The online version contains supplementary material available at 10.1007/s10143-026-04221-0.

## Introduction

Intraoperative aneurysm rupture (IOR), though relatively uncommon, remains one of the most dreaded intraoperative complications in the surgical management of intracranial aneurysms. It is frequently associated with poor neurological outcomes, increased morbidity, and worsened patient prognosis [1–3]. Historically, intraoperative rupture rates during aneurysm surgery were reported as high as 40–50% prior to the advent of modern microsurgical and anesthetic techniques [4, 5]. Although advances in microsurgical technique, intraoperative monitoring, and hemostatic control have reduced this risk, contemporary series still report IOR rates ranging from 7% to 35%, depending on definitions and aneurysm characteristics [6–10].

The current standard of care for intracranial aneurysms consists of both microsurgical clipping and endovascular intervention, with procedural selection guided by aneurysm size, morphology, and patient-specific factors [11]. Although both modalities carry a risk of intra-procedural rupture, the mechanisms, management, and consequences of IOR during microsurgical clipping are distinct. In the microsurgical setting, IOR can result in abrupt loss of operative control, massive blood loss, difficulty achieving hemostasis and proximal control, injury to the peri-aneurysmal neurovascular anatomy due to blind placement of clips, and potential ischemic injury and brain swelling, secondary to prolonged temporary vessel occlusion and/or hemorrhage from the aneurysm [6, 12].

Despite its clinical relevance, the independent impact of IOR on postoperative functional outcomes, mortality, and long-term disability remains incompletely characterized within the literature. Many existing studies group surgical and endovascular cases together, which obscures the specific intraoperative and prognostic implications of rupture during open microsurgical clipping [13]. Among studies limited to surgical cohorts, there remains variability in how IOR is defined, the timing of IOR during the operation when it occurs, and whether its effect persists after adjusting for preoperative clinical grade [9, 14].

Aneurysm size remains a key determinant in surgical planning and risk stratification, yet its relationship to post-rupture outcomes has been inconsistently demonstrated. Larger aneurysms may present greater technical challenges, while smaller aneurysms may rupture under minimal manipulation, complicating efforts to predict prognosis following IOR [15, 16].

Given these inconsistencies, we conducted a systematic review and meta-analysis focused exclusively on surgically treated intracranial aneurysms to quantify the association between IOR and postoperative neurological outcomes, including mortality. A secondary objective was to determine whether aneurysm size influences prognosis following IOR within this surgical cohort.

## Materials and methods

**Fig. 1:**
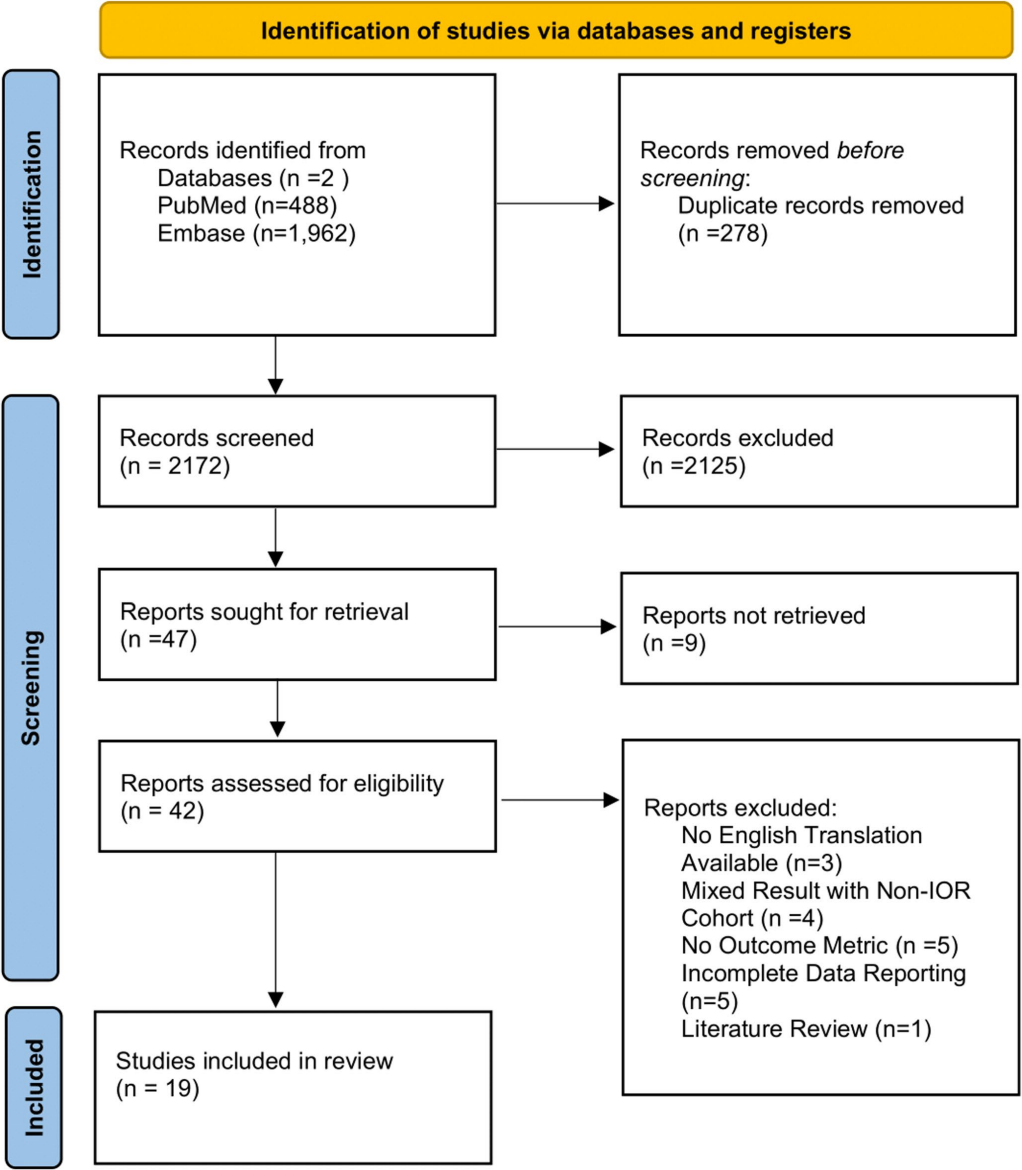
PRISMA flowchart of systematic search for outcomes following intraoperative rupture of cerebral aneurysms during surgical intervention

A systematic literature search of PubMed and Embase was conducted in accordance with PRISMA guidelines to identify studies published between 2000 and June 2025 (Fig. 1). These references were reviewed by two independent reviewers. The search string criteria included the following terms in their respective databases:

- (“Intracranial Aneurysm“[Mesh] OR “cerebral aneurysm*” OR “brain aneurysm*” OR “intracranial aneurysm*”) AND (“intraoperative rupture*” OR “intraoperative complication*” OR “surgical rupture*” OR “intraoperative aneurysm* rupture*” OR “perioperative rupture*” OR “operative rupture*”) AND (“outcome*” OR “prognosis” OR “mortality” OR “morbidity” OR “treatment outcome“[Mesh] OR “survival” OR “neurological outcome*” OR “functional outcome*”)

The search identified 2,450 records; after removal of 278 duplicates, 2,172 records were screened.

We assessed the quality of the articles, the study type, and patient outcomes. Studies were included if they examined microsurgical clipping and reported functional outcomes using the modified Rankin Scale (mRS) or Glasgow Outcome Scale (GOS), with IOR reported as a distinct cohort when applicable. Studies that reported on adult and/or pediatric populations were included in our review.

Studies were excluded if no English translation was available (*n* = 3), IOR was not reported as a distinct cohort (*n* = 4), outcome metrics were absent (*n* = 5), data were incomplete (*n* = 5), or the article was a review (*n* = 1). After exclusions, 19 studies were included, of which 14 provided paired IOR and non-IOR data for quantitative synthesis (Fig. 1).

Extracted data included patient demographics, aneurysm characteristics (location and size), presentation status (ruptured or unruptured), outcome timing, mRS and/or GOS scores, rates of poor outcome, mortality, and complications. When a study reported on paired IOR versus non-IOR cohorts, data were extracted separately for each group. For ruptured aneurysms, baseline clinical and radiographic severity was variably reported using the Hunt–Hess, WFNS, and modified Fisher scales.

Pooled risk ratios (RRs) for poor functional outcome and mortality were calculated using DerSimonian–Laird random-effects models comparing IOR versus non-IOR cohorts and, within IOR cases, SAH versus unruptured presentations. Single-arm pooled proportions of poor outcome among IOR cases were estimated overall and stratified by aneurysm size (< 7 mm, 7–12 mm, 13–24 mm, ≥ 25 mm) and location using logit-transformed random-effects models. Ophthalmic and anterior choroidal arteries were mapped to the internal carotid artery (ICA) category. Heterogeneity was assessed using Cochran’s Q, τ², and I² statistics, with 95% confidence intervals generated around pooled estimates. Pre-specified sensitivity analyses included alternative effect measures and leave-one-out influence diagnostics. Era-based subgroup analyses (pre-2010 vs. 2010 onward) were performed for poor outcomes and mortality. All analyses were conducted in Python 3.11 using open-source packages. ROBINS-1 V2 Bias assessment was completed and located in Table S1. The original dataset and analysis code are available upon reasonable request from the corresponding author. This study was not registered with PROSPERO.

## Results

### Overview

Across 19 studies, 925 patients experiencing intraoperative rupture (IOR) were identified, encompassing 1008 aneurysms [17–35]. Demographic and aneurysm characteristics were extracted and stratified by presentation (SAH vs. unruptured) when available (Table 1). Five studies reported only aggregated cohort characteristics and therefore contributed solely to the total cohort in Table 1.

Among studies reporting stratified data, SAH and unruptured cohorts were demographically similar, with predominantly middle-aged patients and a female predominance, more pronounced in the SAH cohort.

Follow-up duration was generally longer in SAH cohorts than in unruptured aneurysm cohorts. Clinical severity grading using the WFNS and Hunt and Hess scales was reported exclusively for patients presenting with ruptured aneurysms (SAH).

Aneurysm characteristics were variably reported. Where available, most aneurysms measured 7–12 mm, with similar size distributions between ruptured and unruptured cohorts. Anterior circulation aneurysms predominated, most commonly involving the ACoA and MCA.

**Table 1:** IOR demographics based on presentation at baseline

Patients	Ruptured	Unruptured	Total
	555	162	925
Aneurysms	582	162	1008
Median age in yrs (Range)	51.18 (3.30)	52.55 (1.45)	51.35 (12.26)
Median follow up months (IQR)^a^	17.6 (4.5–33.8)	7.3 (3.25-12)	6 (4.5-13.45)
Gender			
M	204 (36.8%)	73 (47.4%)	357 (38.9%)
F	351 (63.2%)	81 (52.6%)	560 (61.1%)
Size			
<7 mm	0 (0.0%)	0 (0.0%)	94 (12.3%)
7–12 mm	344 (96.9%)	154 (95.1%)	599 (67.8%)
13–24 mm	0 (0.0%)	8 (4.9%)	55 (6.0%)
≥ 25 mm	11 (3.1%)	0 (0.0%)	16 (1.7%)
WFNS grade			
I-III	259 (71.0%)		293 (84.9%)
IV-V	106 (29.0%)		129 (15.1%)
HH grade			
I-III	144 (68.6%)		220 (74.1%)
IV-V	66 (31.4%)		77 (25.9%)
Aneurysm site			
ACoA^b^	153 (30.4%)	81 (50.0%)	294 (32.2%)
MCA^c^	131 (26.0%)	44 (27.2%)	278 (30.4%)
ICA^d^	58 (11.5%)	23 (14.2%)	112 (12.3%)
ACA^e^	42 (8.3%)	0 (0.0%)	64 (7.0%)
PCA^f^	2 (0.39%)	0 (0.0%)	2 (0.22%)
PCoA^g^	21 (4.2%)	0 (0.0%)	45 (4.9%)
PICA^h^	3 (0.59%)	1 (0.6%)	6 (0.66%)
Basilar	2 (0.39%)	1 (0.6%)	5 (0.55%)
AICA^i^	0 (0.0%)	0 (0.0%)	1 (0.11%)
Unspecified anterior	54 (10.7%)	0 (0.0%)	54 (5.9%)
Unspecified posterior	7 (1.4%)	6 (3.7%)	15 (1.6%)
Other/Unspecified	31 (6.2%)	6 (3.7%)	37 (4.1%)

*a *Interquartile range, *b *Anterior communicating artery, *c *Middle cerebral artery, *d *Internal carotid artery, *e *Anterior cerebral artery, *f *Posterior cerebral artery, *g *Posterior communicating artery, *h *Posterior inferior cerebellar artery, *i *Anteriorinferior cerebellar artery

*Values in the non-ruptured and ruptured columns do not sum to those in the “Total” column, as five included studies reported mixed (non-stratified) demographic data

### Outcome metrics

Outcomes were assessed using the Glasgow Outcome Scale (GOS, *n* = 10, 52.6%) and the modified Rankin Scale (mRS, *n* = 9, 47.4%). Across all IOR cohorts, 660 patients (65.5%) achieved good outcomes (mRS 1–2 or GOS 4–5), while 348 (34.5%) had poor outcomes (mRS 3–6 or GOS 1–3).

**Fig. 2:**
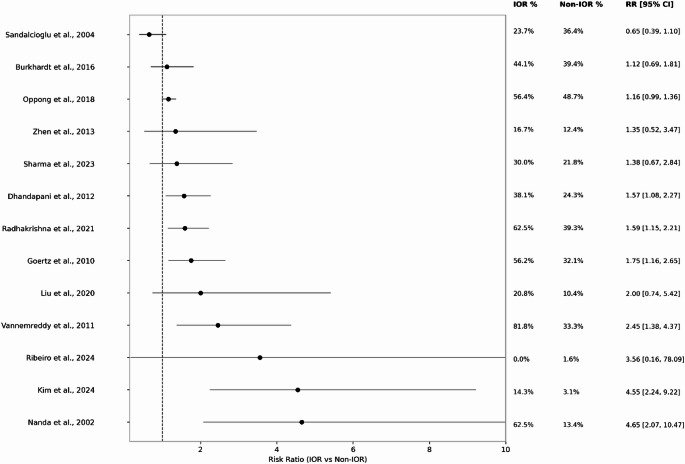
Poor functional outcomes IOR vs. Non IOR, between patients with intraoperative rupture (IOR) and those without IOR (non-IOR). Each of the 14 included studies are shown with its individual risk ratio (RR) and 95% confidence interval, and proportion

**Fig. 3:**
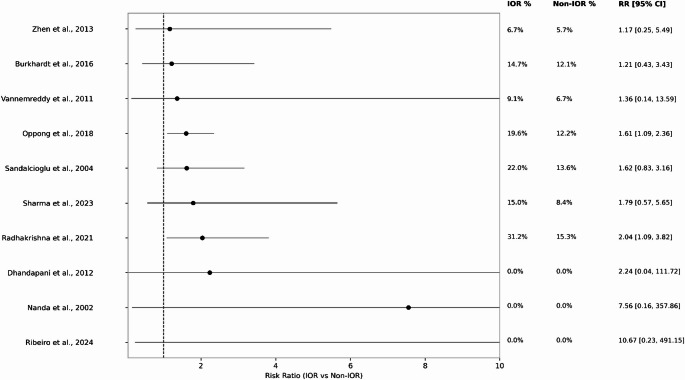
Mortality IOR vs. Non-IOR, between patients with intraoperative rupture (IOR) and those without IOR (non-IOR). Each of the 10 included studies are shown with its individual risk ratio (RR) and 95% confidence interval, and proportion

Fourteen studies reported paired IOR and non-IOR outcomes. Random-effects meta-analysis demonstrated higher risks of poor functional outcome and mortality with IOR [18, 19, 21, 22, 24, 25, 27–31, 35]. For poor functional outcome, the pooled RR was 1.67 (95% CI 1.34–2.09; *p* = .000007; I² = 59.6%; k = 13; Fig. 2). Ten studies reported mortality data, yielding a pooled RR of 2.11 (95% CI 1.37–3.25; I² = 42.8%; k = 11; Fig. 3); zero-event studies were incorporated using continuity correction [18, 19, 21, 22, 24, 25, 27, 29, 30, 32].

Within the IOR cohort, SAH presentation was associated with higher risks of poor functional outcome and mortality compared with unruptured aneurysms. For poor functional outcomes, the pooled RR was 2.01 (95% CI 1.49–2.70; *p* = .00000023). Pooled RR for mortality after IOR was 3.07(95% CI 1.51–6.25; *p* = .00075).

We performed sensitivity checks and subgroup analyses to assess the robustness of the association between IOR and poor outcomes. Across effect measures, results were consistent: RR 1.67 (95% CI 1.34–2.09; I² = 59.6%; *p* = .000007), OR 2.13 (95% CI 1.51–3.00; I² = 51.6%; *p* = .000017), and reduction difference (RD) 0.13 (95% CI 0.07–0.20; I² = 51.6%; *p* = .000053), all indicating higher risk with IOR. Sensitivity analyses using alternative effect measures and continuity corrections yielded nearly identical pooled estimates, confirming result stability.

In single-arm pooling of IOR cohorts, the estimated proportion of poor functional outcome was 0.33 (95% CI 0.26–0.42; I² = 83.8%; k = 19), indicating substantial between-study heterogeneity. This estimate remained robust under Freeman–Tukey transformation (0.34; 95% CI 0.26–0.42). Pooled mortality among IOR cohorts was 0.15 (95% CI 0.11–0.20; I² = 54.5%; k = 16), with a similar estimate by Freeman–Tukey (0.14; 95% CI 0.09–0.19). Overall, approximately one-third of patients with IOR experienced poor functional outcomes and one in seven died, with substantial variability across studies.

### Subgroup analysis

**Fig. 4:**
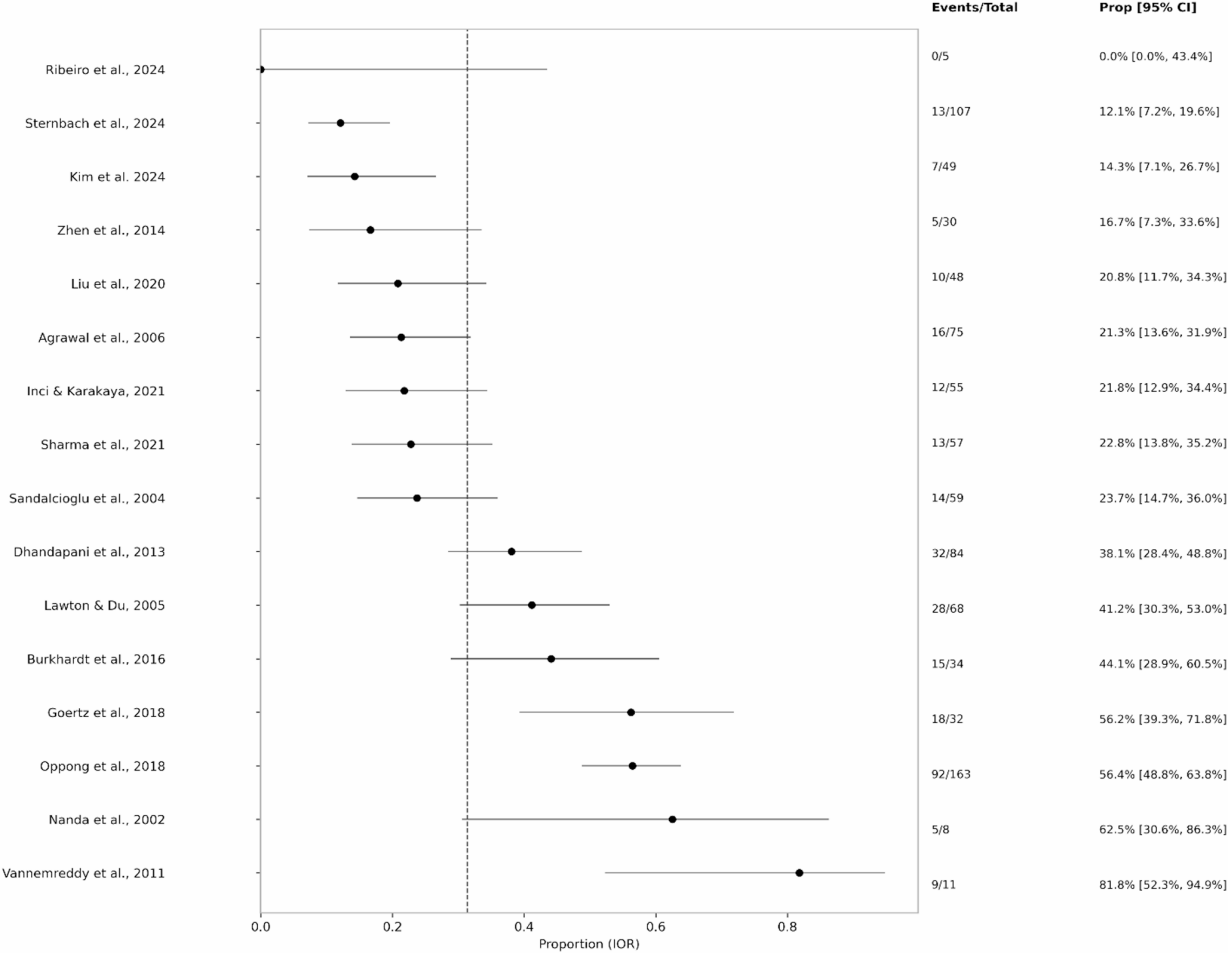
Size pooled poor outcome proportion per study

Size-stratified pooling among IOR cohorts suggested increasing poor-outcome risk with larger aneurysm size where stable estimation was possible. For aneurysms < 7 mm, the pooled poor-outcome proportion was 0.152 (95% CI 0.089–0.249; τ² = 0.0000; I² = 0.0%; Q = 0.08; df = k−1; *p* = .775). For aneurysms 7–12 mm, the pooled proportion was 0.293 (95% CI 0.195–0.415; τ² = 0.6082; I² = 88.0%; Q = 75.11; df = k−1; *p* < .001). For strata of 13–24 mm and ≥ 25 mm, there were insufficient independent cohorts for stable DL estimation; however, study-level and meta-regression analyses indicated an increasing risk of poor outcomes with larger aneurysm size (Fig. 4).

Among pooled analyses by location within IOR cohorts, MCA and ACoA aneurysms demonstrated the highest poor-outcome proportions, 37.4% (95% CI 32.2–42.9%) and 36.2% (95% CI 31.2–41.6%), respectively, followed by aneurysms of ICA (31.5%), posterior communicating artery (PCoA) (26.9%), and anterior cerebral artery (ACA) (25.0%). Mortality proportions were similar across locations (15–15.3%). Formal meta-analytic comparisons by aneurysm location were not possible due to inconsistent reporting.

Era subgrouping suggested attenuation of poor outcome and mortality in more contemporary series: pre-2010, RR 1.88 (95% CI 0.31–11.43; I² = 93.0%); 2010+, RR 1.65 (95% CI 1.39–1.97; I² = 31.5%), with a significant between-subgroup difference (Q_between = 4.01; df = 1; *p* = .045).

## Discussion

To our knowledge, this is the first systematic review and meta-analysis focused exclusively on surgically clipped cerebral aneurysms evaluating the impact of intraoperative rupture (IOR) on neurological outcomes. Across 19 studies involving 925 patients, IOR was associated with increased risks of poor functional outcome (RR 1.67) and mortality (RR 2.11), with even higher risks when rupture occurred in the setting of SAH [1–4].

The approximately one-third rate of poor outcomes among patients experiencing IOR highlights the substantial physiological disruption caused by rupture. IOR may occur before or during aneurysm neck dissection, often resulting in abrupt loss of operative control, impaired visualization, and an increased risk of ischemic injury or direct vessel damage.

When a rupture occurs prior to the establishment of proximal and distal control, the hemorrhage could continue for a relatively long period of time, leading to increased intracranial pressure and subsequent cerebral swelling, which may lead to further neurological injury [6, 9, 12]. Such rupture prior to any apparent aneurysm manipulation is much more common with previously ruptured aneurysms, which is inherently fraught with complications. Surgical maneuvers such as brain retraction or parent vessel manipulation may precipitate such rupture, resulting in prolonged hemorrhage, cerebral swelling, and ischemic injury.

### Aneurysm characteristics and intraoperative rupture

Pooled analyses suggested a trend toward higher poor-outcome rates with increasing aneurysm size; however, limited data in larger size strata preclude firm conclusions. Similarly, MCA and ACoA aneurysms showed slightly higher pooled proportions of poor outcomes than other locations, but these represent associations rather than definitive causal effects. These trends may reflect differences in anatomical accessibility, surgical exposure, and timing of proximal control, though independent effects of location cannot be determined [10, 11].

### Other factors

Era-based analyses suggested attenuation of risk in contemporary series, consistent with advances in surgical exposure, temporary clipping, and anesthetic management [9, 14, 16]; however, IOR remained significantly associated with poor outcomes. Sensitivity analyses using alternative effect measures and continuity corrections confirmed the robustness of these associations.

The high heterogeneity across studies likely reflects variability in patient characteristics, aneurysm morphology, surgeon experience, and institutional protocols [1, 9, 13]. Multivariate analyses identified aneurysm complexity, morphology, and poor preoperative clinical grade as predictors of poor outcome after IOR, underscoring the multifactorial nature of risk [12, 15, 16]. These findings highlight the importance of integrating patient-, aneurysm-, and procedure-level factors into preoperative risk assessment and intraoperative decision-making [36–38].

### Prevention and management of intraoperative rupture

While refinements in microsurgical techniques, anesthetic management, and temporary clipping strategies have reduced the frequency and severity of IOR over the past decades [6, 7, 12, 14], its association with adverse outcomes persists, indicating that these advances have mitigated but not eliminated the clinical consequences of rupture.

Therefore, the primary strategy for managing IOR is to make every effort to prevent it, as our results demonstrate that IOR can negatively impact patient outcomes, particularly in patients with SAH. Proper patient positioning, gravity-assisted retraction, and meticulous subarachnoid dissection to achieve a relaxed brain are essential, and forceful retraction should be avoided. Adequate proximal control via subarachnoid dissection facilitates safe temporary clip placement and may require skull base drilling or cervical ICA exposure. Testing of the feasibility of the proximal temporary clip placement before proceeding to the dissection of the aneurysm is of paramount importance. Following the proximal and distal control of the peri-aneurysmal vessels, the aneurysm neck should be dissected. Dissection of the aneurysm dome should be postponed to the very last moments of aneurysm preparation. A large, tense aneurysm may require complete trapping to make it softer and more easily clippable. The suction decompression technique for certain ICA aneurysms is a useful way to facilitate aneurysm dissection and clipping [39]. The surgeon must determine the optimal timing for temporary clipping, ensuring it is neither too early, which could lead to excessive ischemia, nor too late, as the fragile aneurysm wall may rupture before the parent vessel(s) are temporarily clipped. A tear in the neck of the aneurysm is a dreaded complication that may be difficult to manage [40, 41].

Unfortunately, despite taking all necessary precautions, an IOR may still occur. All the maneuvers mentioned above are part of a contingency plan in case of an IOR. Therefore, it is essential to anticipate this possibility and ensure that everything is properly prepared to manage it effectively. Additional measures may be taken to further facilitate the management of an IOR should it happen. A large craniotomy in cases of a previously ruptured aneurysm helps mitigate the impact of brain swelling in case of an IOR. Having an external ventricular drain to control the rapid rise of the intracranial pressure (or at least having prepared the cranial area for a ventriculostomy beforehand), coordination with anesthesia and neuromonitoring teams for optimizing blood pressure and pharmacologic neuroprotection, as well as preparation for administration of adenosine to temporarily pause the hemorrhage while a temporary clip is being applied to the parent vessel increase the seamlessness of managing IOR. The surgeon should avoid the temptation to blindly tamponade the bleeding area and instead try to use a combination of suction and tamponade of the bleeding to locate the proximal parent vessel to apply a temporary clip. With proper suctioning of the blood, the window opens for establishing proximal control and placing a temporary clip on the parent vessel. Having three suction tubes in the field helps to rapidly switch out any nonfunctioning suction tip. Cross-matched blood should be available in the room at the start of the case to prevent loss of control due to massive blood loss during the bleeding time.

Typically, the same strategies could be applied when the aneurysm ruptures during the dissection of its neck. However, in such a case, the proximal and distal vessel controls have already been established, and application of temporary clips to shut off the blood flow into the aneurysm should be facilitated. If brisk back-bleeding persists, a distal clip should be placed when necessary. It is reasonable to believe that an IOR, occurring before achieving both proximal and distal control, is more likely to lead to poor outcomes. Therefore, all efforts should be made to reduce the risk of an early IOR.

These findings underscore the importance of meticulous preoperative planning and intraoperative strategies, including assessment of aneurysm morphology, judicious temporary clipping, and rapid proximal control, to mitigate the consequences of rupture [6, 12, 15, 16, 27, 28, 42]. One could argue that a properly timed temporary clipping of the parent artery would provide a beneficial outcome, as post-rupture proximal and distal control may eventually lead to a longer ischemia time due to the compounded complexity of the situation. Awareness of potential associations with aneurysm size and location can guide intraoperative vigilance but should not be overinterpreted as definitive predictors of outcome. Patient counseling should incorporate the spectrum of potential outcomes following IOR, supporting informed decision-making and realistic expectations [1, 3, 29, 43].

### Study limitations

Limitations of this meta-analysis include heterogeneity in the definition and timing of IOR, incomplete reporting of baseline clinical grades, and the predominance of retrospective single-center studies, which limit the generalizability of the findings. Additionally, few studies reported multivariate-adjusted effect estimates, leaving the possibility of residual confounding [1, 9, 13, 14]. Despite these constraints, sensitivity analyses demonstrated consistent directionality and magnitude of effect across study subgroups.

### Future directions

Future research should prioritize the development of standardized definitions for IOR and the systematic reporting of functional outcomes. Prospective, multicenter studies would improve generalizability and provide a stronger basis for risk modeling. Advanced intraoperative monitoring techniques such as indocyanine green angiography, neuromonitoring, and continuous hemodynamic assessment may help prevent or respond to rupture events more rapidly [44, 45]. Integrating patient-specific factors, aneurysm morphology, and surgeon experience into predictive models could inform individualized operative strategies [46, 47]. Collaborative registries that capture both intraoperative events and long-term functional outcomes would further strengthen evidence-based approaches to aneurysm surgery [48, 49].

## Conclusion

IOR during microsurgical clipping of cerebral aneurysms remains a significant predictor of adverse neurological outcomes and mortality. Despite advances in surgical technique, anesthetic management, and intraoperative monitoring, IOR continues to pose substantial risk, with approximately one in three patients experiencing poor functional outcomes and a doubling of mortality compared with non-rupture cases. While pooled analyses suggest trends toward higher risk with larger aneurysms and certain locations, these associations should be interpreted cautiously due to study heterogeneity and limited data in specific subgroups.

The persistence of poor outcomes following IOR of aneurysms underscores the importance of meticulous preoperative planning, intraoperative vigilance, and development of strategies to minimize the risk of IOR, and rapid and standardized IOR management strategies. Standardization of IOR definitions, systematic reporting of functional outcomes, and prospective multicenter studies are necessary to enhance risk stratification, optimize intraoperative techniques, improve patient counseling, and ultimately improve prognosis for patients undergoing microsurgical aneurysm repair.

## Supplementary information

Below is the link to the electronic supplementary material.

Supplementary Material 1

Supplementary Material 2

## Data Availability

The original dataset and analysis code are available upon reasonable request from the corresponding author.
