# Supplementary material for: Outcomes following intraoperative rupture of cerebral aneurysms during microsurgical clipping: a systematic review and meta-analysis
Source: Neurosurg Rev. 2026 Mar 18;49(1):290. doi: 10.1007/s10143-026-04221-0 (PMC12999670; doi:10.1007/s10143-026-04221-0)
Supplement: Supplementary file 1 — Supplementary Material 1 [file 10143_2026_4221_MOESM1_ESM.docx]

**Outcomes Following Intraoperative Rupture of Cerebral Aneurysms during Microsurgical Clipping: A Systematic Review and Meta-Analysis**

Neurosurgical Review

Brooklyn Brekke-Kumley, BS^1^; Kiana Yeganeh, BS^2^; Mackenzie Fox, BS^1^; Kristin Cler, BS^1^; Michael T. Lawton, MD^3^, Ali Tayebi Meybodi, MD ^4*^

1. Rocky Vista University, Montana College of Osteopathic Medicine; Billings, MT, USA

2. Ponce Health Sciences University, School of Medicine; Ponce, Puerto Rico

3. Department of Neurosurgery, Barrow Neurological Institute, Phoenix, Arizona, USA

4. Department of Neurological Surgery, Rutgers- New Jersey School of Medicine, Newark, NJ, USA

**Correspondence:**

**Ali Tayebi Meybodi, MD**

Department of Neurological Surgery,

Rutgers New Jersey School of Medicine,

Newark, NJ 08901, USA.

Email: [Tayebi.a77@gmail.com](mailto:Tayebi.a77@gmail.com)

**Table S2. Summary of Included Studies**

| Investigator | Design | Era | Sample size | Aneurysms included | Location | Size metric | Outcome scale | Follow-up time | Poor Outcome | Mortality |
| --- | --- | --- | --- | --- | --- | --- | --- | --- | --- | --- |
| Lawton & Du., 2005 [17] | Retrospective | 2000-2009 | 906 patients; 1038 aneurysms | 60 subarachnoid hemorrhage, 8 unruptured | 23 AComA, 14 PComA, 11 MCA, 2 Opthalmic Artery, 2 Anterior choroidal, 5 ICA, 1 pericallosal, 4 basilar apex, 1 anteroinferior cerebellar, 5 posterior inferior cerebellar | NR | GOS | 4.5 mo | 28 | 12 |
| Nanda, et al 2002 [18] | Retrospective | 2000-2009 | 75 patients; 78 aneurysms | 8 Unruptured | 4 ICA, 1 Basilar, 1 PICA, 2 MCA | 20.9 (8-26) (SD 7.9) | GOS | 6 mo | 5 | 0 |
| Sandalcioglu et al., 2004 [19] | Retrospective | 2000-2009 | 169 patients | 59 subarachnoid hemorrhage | 27 ACA, 15 ICA, 16 MCA, 1 PC | NR | GOS | 6 mo | 14 | 13 |
| Agrawal, 2006 [20] | Retrospective | 2000-2009 | 351 patients | 75 unruptured | 75 AcomA | 60 (5-10) 15 (11-25) | GOS | 12 mo | 16 | 6 |
| Dhandapani et al., 2013 [21] | Prospective cohort | 2010-2019 | 273 patients | 84 subarachnoid hemorrhage | 21 MCA, 9 DACA, 37 AcomA, 17 ICA | 79 (<12) 4 (13-24) 1 (>25) | GOS | 3 mo | 32 | 0 |
| Zhen et al., 2014 [22] | Retrospective | 2010-2019 | 135 patients; 148 aneurysms | 31 unruptured | 15 AComA, 8 PComA, 3 MCA, 3 ACA, 2 ICA | 4.73 (1.6-18.23) | GOS | 6 mo | 5 | 2 |
| Sternbach et al., 2024 [23] | Retrospective | 2010-2019 | 150 patients; 156 aneurysms | 5 subarachnoid hemorrhage, 1 unruptured | 156 MCA  (139 M1, 9 M2, 3 M3, 1 M4) [for total cohort] | 7.4 +/- 5.9 [for total cohort] | mRS | 14.9 mo | 13 | 6 |
| Sharma et al., 2024 [24] | Retrospective | 2020+ | 199 patients; 251 aneurysms | 20 Subarachnoid hemorrhage | AComm: 8  MCA: 4  PComm: 2  ICA: 1  DACA: 2  PICA: 2  PCA: 1  Basilar tip: 0 | NR | mRS | 58 mo | 6 | 3 |
| Vannemreddy et al., 2011 [25] | Retrospective | 2010-2019 | 41 patients | NR | Ophthalmic artery: 4  ICA: 2  MCA: 2  Basilar: 2  PICA: 1 | > 25 mm (all giant aneurysms) | GOS | 50 mo | 9 | 1 |
| Lakićević et al., 2015 [26] | Retrospective | 2010-2019 | 747 patients | 84 subarachnoid hemorrhage | NR | NR | mRS | NR | 33 | 15 |
| Burkhardt et al., 2016 [27] | Retrospective | 2010-2019 | 100 patients | 34 Subarachnoid hemorrhage | 15 MCA, 12 AcomA, 5 ICA, 2 PComA | 9.2 (+-7.2) | GOS | 17.6 mo | 15 | 5 |
| Goertz, et al., 2018 [28] | Retrospective | 2010-2019 | 138 patients | 36 subarachnoid hemorrhage | 30 AComA, 1 distal ACA, 52 MCA, 11 PComA, 8 ICA, 4 post circulation | 8.3 +/- 3.5 | mRS | 6 mo | 18 | NR |
| Ribeiro et al., 2024 [29] | Retrospective | 2020+ | 68 patients | 5 unruptured | 5 MbifAs (main bifurcation aneurysms) | 7.9 +/- 3.4 | mRS | 12 mo | 0 | 0 |
| Oppong et al., 2018 [30] | Retrospective | 2010-2019 | 711 patients; 903 aneurysms | 137 subarachnoid hemorrhage, 26 unruptured | 68 MCA, 58 AComA | 7.45 +/- 5.09 | mRS | 6 mo | 92 | 32 |
| Liu et al., 2020 [31] | Retrospective | 2020+ | 96 patients | 48 unruptured | 19 ICA, 19 MCA, 4 AComA, 6 PC | <7 (15), 7-12 (28), 13-24(5) | mRS | 0.5 mo | 10 | NR |
| Radhakrishna et al, 2021 [32] | Retrospective | 2020+ | 195 patients; 227 aneurysms | 41 subarachnoid hemorrhage | 15 AComA, 3 ACA, 11 MCA, 6 PCoA, 6 ICA | NR | GOS | 0 mo | 20 | 10 |
| Inci & Karakaya, 2021 [33] | Retrospective | 2020+ | 775 patients; 1000 aneurysms | 50 subarachnoid hemorrhage, 5 unruptured | 9 ICA [3 paraclinoid, 4 PCommA, 1 anterior choroidal artery, 1 bifurcation], 19 MCA [19 bifurcation], 25 Acom A [1 pericallosal], 1 posterior circulation | <10 (30), 10-25 (21), >25 (4) | mRS | 37.8 mo | 12 | 7 |
| Sharma, et al 2021 [34] | Retrospective | 2020+ | 471 patients | 57 Subarachnoid hemorrhage | 54 anterior, 3 posterior | >10mm 10, < 10 mm 47 | GOS | 12 mo | 13 | NR |
| Kim et al 2024 [35] | Retrospective | 2020+ | 4027 patients; 4282 aneurysms | 30 unruptured 19 subarachnoid hemorrhage | 20 ACA [19 ACOMA], 17 ICA [12 PCOMA], 22 MCA, 2 PCA | 4.95 (3.85-6.51) | mRS | 3 mo | 7 | 11 |
