# Supplementary material for: Outcomes following intraoperative rupture of cerebral aneurysms during microsurgical clipping: a systematic review and meta-analysis
Source: Neurosurg Rev. 2026 Mar 18;49(1):290. doi: 10.1007/s10143-026-04221-0 (PMC12999670; doi:10.1007/s10143-026-04221-0)
Supplement: Supplementary file 2 — Supplementary Material 2 [file 10143_2026_4221_MOESM2_ESM.docx]

**Outcomes Following Intraoperative Rupture of Cerebral Aneurysms during Microsurgical Clipping: A Systematic Review and Meta-Analysis**

Neurosurgical Review

Brooklyn Brekke-Kumley, BS^1^; Kiana Yeganeh, BS^2^; Mackenzie Fox, BS^1^; Kristin Cler, BS^1^; Michael T. Lawton, MD^3^, Ali Tayebi Meybodi, MD ^4*^

1. Rocky Vista University, Montana College of Osteopathic Medicine; Billings, MT, USA

2. Ponce Health Sciences University, School of Medicine; Ponce, Puerto Rico

3. Department of Neurosurgery, Barrow Neurological Institute, Phoenix, Arizona, USA

4. Department of Neurological Surgery, Rutgers- New Jersey School of Medicine, Newark, NJ, USA

**Correspondence:**

**Ali Tayebi Meybodi, MD**

Department of Neurological Surgery,

Rutgers New Jersey School of Medicine,

Newark, NJ 08901, USA.

Email: [Tayebi.a77@gmail.com](mailto:Tayebi.a77@gmail.com)

**Table S1. ROBINS-I V2 Bias Assessment**

| Study | Bias due to confounding | Bias in selection of participants | Bias in classification of interventions | Bias due to deviations from intended interventions | Bias due to missing data | Bias in measurement of outcomes | Bias in selection of reported results | Overall bias |
| --- | --- | --- | --- | --- | --- | --- | --- | --- |
| Lawton & Du, 2005 [17] | Moderate | Low | Low | Moderate | Low | Low | Moderate | Moderate |
| Nanda et al., 2002 [18] | Low | Low | Low | Low | Low | Low | Low | Low |
| Sandalcioglu et al., 2004 [19] | Moderate | Moderate | Low | Moderate | Moderate | Low | Moderate | Moderate |
| Agrawal et al., 2006 [20] | Moderate | Moderate | Moderate | Moderate | Moderate | Moderate | Serious | Serious |
| Dhandapani et al., 2013 [21] | Low | Low | Low | Low | Low | Low | Low | Low |
| Zhen et al., 2014 [22] | Moderate | Low | Low | Moderate | Moderate | Low | Moderate | Moderate |
| Sternbach et al., 2024 [23] | Serious | Moderate | Moderate | Serious | Serious | Moderate | Serious | Serious |
| Sharma et al., 2024 [24] | Low | Low | Low | Low | Low | Low | Low | Low |
| Vannemreddy et al., 2011 [25] | Moderate | Low | Low | Moderate | Low | Low | Moderate | Moderate |
| Lakicevic et al., 2015 [26] | Low | Low | Low | Low | Low | Low | Low | Low |
| Burkhardt et al., 2016 [27] | Moderate | Low | Low | Moderate | Moderate | Low | Moderate | Moderate |
| Goertz et al., 2018 [28] | Low | Low | Low | Low | Low | Low | Low | Low |
| Ribeiro et al., 2024 [29] | Moderate | Moderate | Moderate | Serious | Moderate | Moderate | Serious | Serious |
| Oppong et al., 2018 [30] | Low | Low | Low | Low | Low | Low | Low | Low |
| Liu et al., 2020 [31] | Moderate | Moderate | Low | Moderate | Moderate | Low | Moderate | Moderate |
| Radhakrishna et al., 2021 [32] | Moderate | Moderate | Moderate | Moderate | Moderate | Moderate | Serious | Serious |
| Inci & Karakaya, 2021 [33] | Moderate | Low | Low | Moderate | Moderate | Low | Moderate | Moderate |
| Sharma et al., 2021 [34] | Low | Low | Low | Low | Low | Low | Low | Low |
| Kim et al. 2024 [35] | Moderate | Moderate | Low | Moderate | Moderate | Low | Moderate | Moderate |
